# Integrated analysis of dosage effect lncRNAs in lung adenocarcinoma based on comprehensive network

**DOI:** 10.18632/oncotarget.19864

**Published:** 2017-08-03

**Authors:** Yunzhen Wei, Zichuang Yan, Cheng Wu, Qiang Zhang, Yinling Zhu, Kun Li, Yan Xu

**Affiliations:** ^1^ College of Bioinformatics Science and Technology, Harbin Medical University, Harbin, China

**Keywords:** SCNA, dosage effect, lncRNAs, comprehensive network, lung adenocarcinoma

## Abstract

Accumulating evidences indicate that cancer-related lncRNAs occur frequent somatic copy number alternation (SCNA). Although individual SCNA lncRNAs have been implicated in tumor biology, their regulatory mechanism has not been assessed in a systematic way. In order to explore the expression characteristics and biological functions of SCNA lncRNAs in cancer, we built a computational framework based on lncRNA expression profiles, lncRNA copy numbers and dosage sensitivity score (DSS). First, we found that the lncRNAs with different DSS were involved in distinct biological processes, while those with the same DSS had similar functions. Second, some of the lncRNAs participated in the progression and metastasis of lung adenocarcinoma (LUAD) through cis-acting regulation. In lncRNA-TF-mRNA network, lncRNAs interacted with 4 TFs and affected the immune system, and further influenced LUAD progression. Third, competing endogenous RNA network analysis inferred that lncRNA ENSG00000240990 competed with HOXA10 to absorb hsa-let-7a/b/f/g-5p and affected patient prognosis in LUAD. Last but not least, by integrating target information of miRNA we also provided a new perspective for the discovery of potential small molecule drugs. In summary, we systematically analyzed the regulatory role of SCNA lncRNAs. This work may facilitate cancer research and serve as the basis for future efforts to understand the role of SCNA lncRNAs, develop novel biomarkers and improve knowledge of tumor biology.

## INTRODUCTION

Non-small cell lung cancer (NSCLC), which accounts for 70-80 percent of lung cancer, is one of the leading causes of death world-wide and is notoriously difficult to treat effectively. Particularly, lung adenocarcinoma (LUAD) is the most common subtype of NSCLC [1, 2]. The success of cancer treatment depends on the early examination and diagnosis. However, many patients are diagnosed with cancer in metastasis or terminal period. Though the technology of molecular diagnosis and target therapy is improving, the overall survival of LUAD patients in 5 years is low [3]. Therefore, it is imperative for clinic to identify suitable molecular markers for LUAD diagnosis and prognosis.

The high-throughput sequencing analysis revealed that the non-coding RNA made up the preponderance of genome and one of the subsets, the lncRNA, was proved to affect tumor development and pathology through chromosome modification, transcription and post-transcriptional process [4]. For example, lncRNA MALAT1 regulated the expression of genes that were associated with lung cancer metastasis [2]; lncRNA CCAT1 acted as an oncogene and promoted chemoresistance in docetaxel-resistant LUAD cells [1]. Recently, a long intergenic non-coding RNA (lincRNA) p21 was identified as a mediator and a key target of p53 DNA damage response [5, 6].

Furthermore, cancer is a kind of genetic disease that is related with multiple variations of genome. Davoli *et al.* found that the cancer driver genes tended to have SCNA in genome, and the frequent alterations of DNA copy number promoted cancer progression [7]. Actually, SCNA was verified to be associated with various cancers such as LUAD, breast cancer and liver cancer [8–10].

In fact, SCNA affects cancer progression in an indirect way and contributes to pathway disorder by regulating gene expression. Previous studies showed that the dosage sensibility of SCNA was closely related to cancer [11]. The aberrance of gene expression that induced by SCNA could be described by dosage sensibility [12]. Nevertheless, because of the difference of dosage sensibility, SCNA may not have the same influence on gene expression. The change of dosage sensibility could control the disorder of gene expression. Moreover, some studies indicated that SCNA was a key mechanism for the disorder of cancer-related lncRNAs [13]. For example, PVT1 was found to occur copy number amplifications accompanied by oncogene MYC and promoted the expression of MYC. The MYC expression may return to the precancerous condition through breaking the cooperative relationship between PVT1 and MYC [14]. Evidently, the discovery supported the hypothesis that the dosage effect of lncRNAs had potential functions in cancer. Therefore, it is important to have a deep insight into the lncRNAs that occur SCNA to explain the potential mechanism of disease progression and find accurate treatment of cancer.

Nowadays, only a small group of lncRNAs is known to be related with diseases. Though lacking the ability to code proteins, lncRNAs can regulate the transcription and translation of neighboring genes significantly, leading to a series of biological progressions such as dosage compensation, genomic imprinting and cell cycle control [15]. Typically, many studies described the function of lncRNAs based on the *Guilty by Association* principle [16]. These studies explored the functions of lncRNAs in biological progression and cancer by constructing a co-expression model of protein-coding genes (PCGs) and lncRNAs [16, 17]. For instance, lncRNA MALAT1 participated in LUAD metastasis *via* regulating gene expression [2]. Consequently, with the burning desire to describe the functions of lncRNAs deeply, more and more regulated functions were discovered. First, lncRNAs can regulate gene expression through cis-regulation and trans-regulation function [18, 19]. With cis-regulation function, lncRNAs may influence the expression of neighboring genes [20]. Accumulated evidences demonstrated that part of lncRNAs were positively correlated with the expression level of PCGs [17, 21]. For example, the lncRNA Jpx positively affected the expression of neighboring gene Xist [22]. In addition, recent studies also found that lncRNA was related with TF [23]. Take lncRNA Evf-2 as an example, it could promote the transcription of Dlx gene cluster by forming a stable compound with TF Dlx2 [24]. A novel hypothesis believed that lncRNAs could act as miRNA sponges and regulate gene expression through competing endogenous RNA (ceRNA) mechanism [1, 25]. An increasing number of evidences proved that ceRNA lncRNAs played a key role in cancer occurrence and progression. Xu *et al.* found that the majority of the interactions of miRNA sponges was cancer-specific which formed conserved and cancer-specific models by the interactions among sponges [26]. Nonetheless, seldom do people systemically describe the function and expression characteristics of SCNA lncRNAs. Our study analyzed the genomic characteristics of SCNA lncRNAs based on DSS. Furthermore, we described the functions and regulations of SCNA lncRNAs in cis-regulating network, trans-regulating network and ceRNA network. We constructed an lncRNA-function network and depicted the functions of SCNA lncRNAs in cancer progression. Finally, we identified prognosis-related lncRNAs and nominated potential small molecular drugs for LUAD treatment. Taken together, our study systematically analyzed SCNA lncRNAs, provided a foundation for deeper understanding the role of cancer-related SCNA lncRNAs and improved knowledge of tumor biology.

## RESULTS

### LncRNAs exhibit frequent SCNAs in LUAD

**Figure 1:**
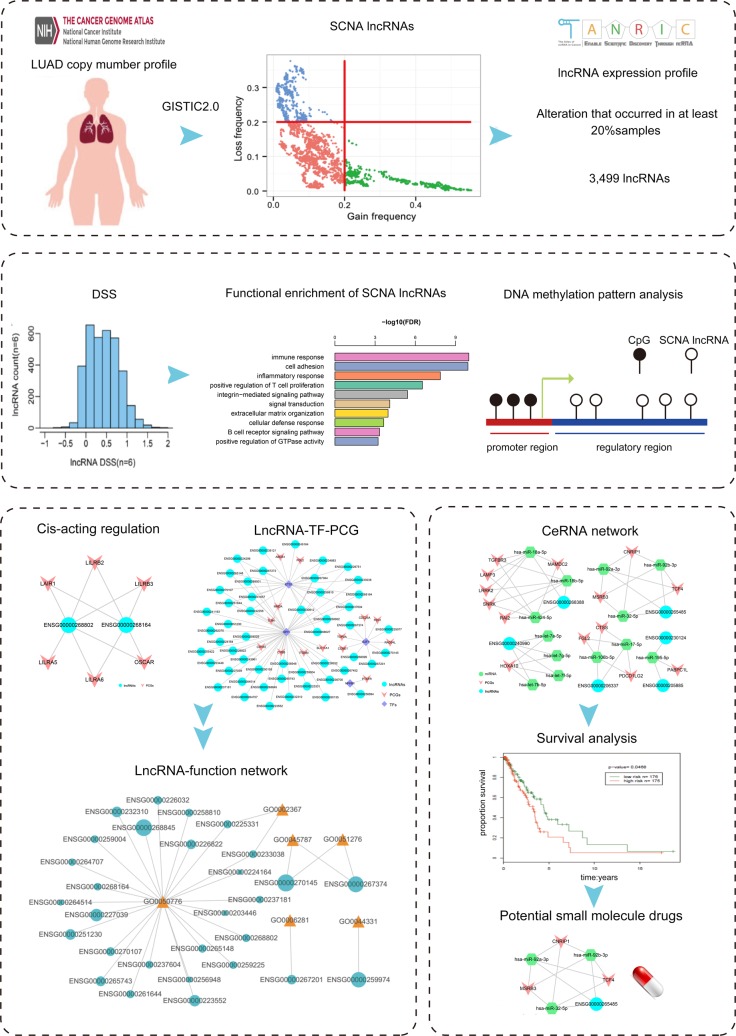
Flowchart of the study **A.** The identification of SCNA lncRNAs. **B.** The calculation of DSS of SCNA lncRNAs and the basic analysis. **C.** and **D.** The construction of cis-acting network, lncRNA-TF-PCG network and ceRNA network.

We proposed a pipeline to systemically analyze the functions of SCNA lncRNAs, and the approach was applied to the LUAD dataset (Figure 1). We analyzed the LUAD copy number profile that was calculated by GISTIC2.0 and identified 128 independent genomic variable fragments (49 gains and 79 losses). Previous study indicated that SCNA was an important mechanism that led to the dysregulation of lncRNAs in cancer, especially for those cancer types whose genomes contained abundant SCNAs [27]. Therefore, we obtained the genomic local information of 13,870 lncRNAs from GENCODE. In order to nominate the lncRNAs that acted as driver genes in tumor occurrence and development, the lncRNAs sites were mapped to the 128 independent genomic variable fragments (Supplementary Figure 1). 429 and 2,923 lncRNAs were mapped to the focal gains and losses fragments, respectively. In total, 1,060 lncRNAs had expression value (179 amplified and 881 deleted lncRNAs, Supplementary Data S1).

### The dosage effect score of lncRNAs

We calculated the copy number frequency of each lncRNA in variable fragments through copy number discretization matrix. The alteration that occurred in at least 20% samples was defined as high-frequency alteration. In fact, few lncRNAs occurred high-frequency gain and loss at the same time. As a result, 3,499 lncRNAs had high-frequency alterations, most of which were intergenic and trans-lncRNAs (Supplementary Data S2).

**Figure 2:**
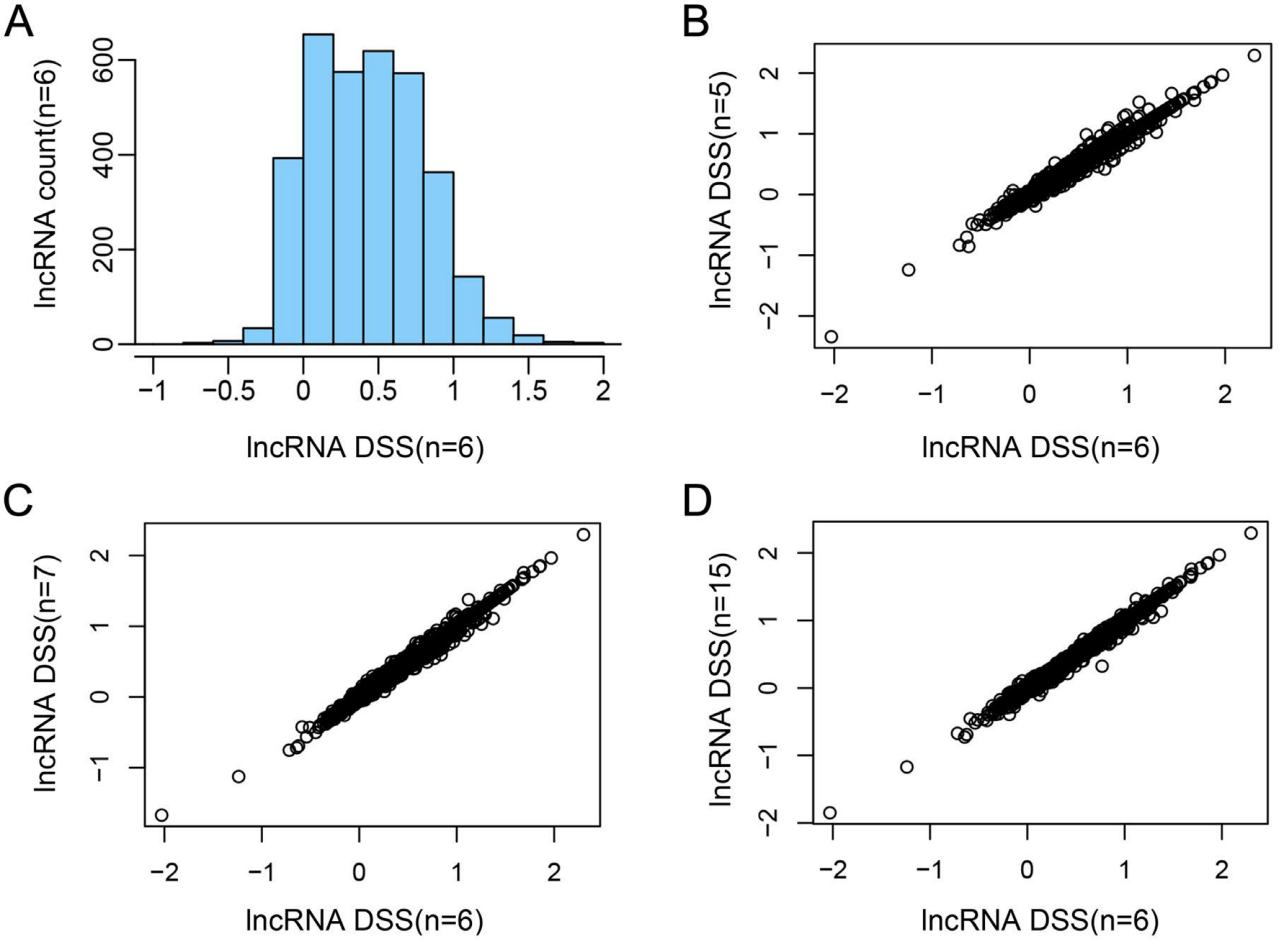
The DSS of lncRNAs in different parameters **A.** The DSS of lncRNAs (*n* = 6). X-axis represents the DSS of each lncRNA, y-axis represents the number of lncRNAs. **B.**-**D.** The comparison of DSS among different parameters (*n* = 5, 6, 7 and 15).

In order to evaluate the impact of SCNA on the deregulation of lncRNAs, we analyzed the association between lncRNA copy number and RNA expression level by calculating DSS [28]. The DSS of lncRNAs was showed in Figure 2A. The result showed that most scores of lncRNAs were low.

In the DSS calculation formula, the parameter n determined the monotonicity score. In order to obtain precise result, we performed parameter selection. The initial parameter n was set as 6. As control, the *n* = 5, 7 and 15 were selected to calculate the DSS of genes (Figure 2B-2D). The distributions of DSS were similar in different parameters, confirming the robust of our approach.

### Enrichment analysis of SCNA lncRNAs

As we know, most of the lncRNAs lack annotation. Their functions are usually assessed by the co-expressed PCGs. Our study identified significant 25,871 lncRNA-PCG pairs (1,133 lncRNAs and 1,976 PCGs) based on the profile of 3,449 SCNA lncRNAs and 3,654 differentially expressed PCGs. We divided 1,133 lncRNAs into 7 groups based on the DSS of lncRNAs and performed functional enrichment analysis for co-expression PCGs of each group (FDR < 0.05).

**Figure 3:**
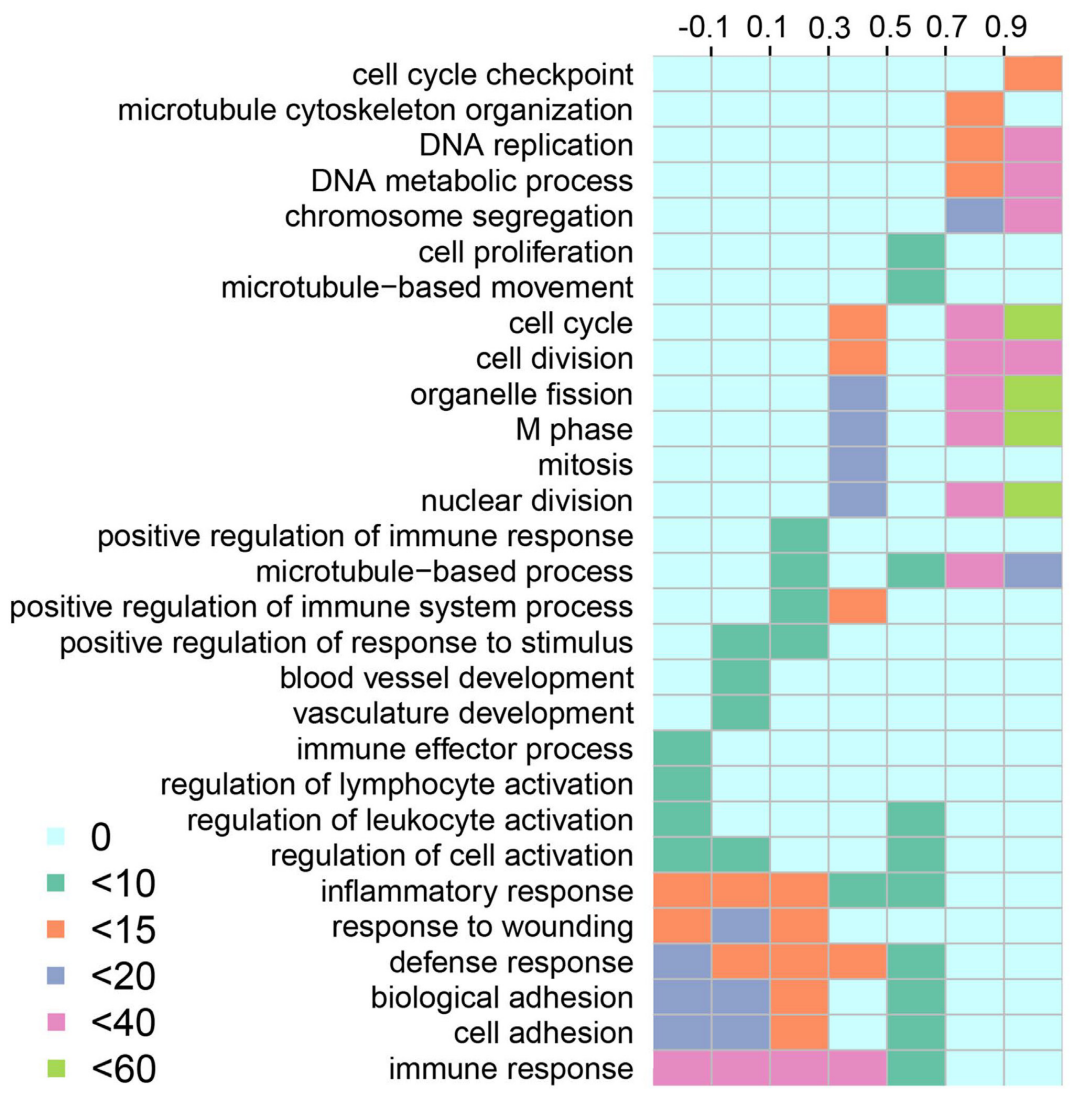
Functional enrichment of SCNA lncRNAs The columns represent the score Intervals of lncRNAs. The rows represent the significant biological processes that enriched by lncRNAs. The different colors represent the degree of the enrichment significance (−log 10 (FDR)).

The result indicated that the low-sensitivity lncRNAs were mainly enriched in immune-associate biology processes, including immunoreaction, inflammatory response, cell adhesion, chemotaxis, cell proliferation, repair in trauma, angiogenesis and cell migration (Figure 3). The immune system was an important mechanism for immune response and had positive effects on immunological surveillance, defense and regulatory. Immune system could hinder the escape of cancer cell by specific mechanism. Lung cancer immunization therapy located the target site of cancer cells by using immune checkpoint inhibitors, which contributed to the interruption of misleading signals that emitted by cancer cell and the elimination of cancer cells [29, 30]. Researchers could re-activate the immune cell and inhibit the growth of cancer cell by breaking through the sentinel point. The low-dosage sensitive lncRNAs that identified by our study may provide new perspective for the identification of sentinel point. The functions of SCNA lncRNAs was changed from immune system to some key processes that maintained cell ordering and genomic DNA stability, including cell cycle, cell differentiation, DNA repair, cytoskeleton and chromosome assembly. The disorder of cell cycle regulation was one of the characteristics of cancer cells [31] and the regulation of cell cycle was an elaborate equilibrium process. Nowadays, the researches of various regulatory factors and regulatory mechanisms provide a solid theory foundation for tumor occurrence and development. Therefore, the high-dosage sensitive lncRNAs that we identified may contribute to the further research of cell cycle regulatory factor, and the relationship among them could promote the understanding of oncobiology.

### DNA methylation analysis

**Figure 4:**
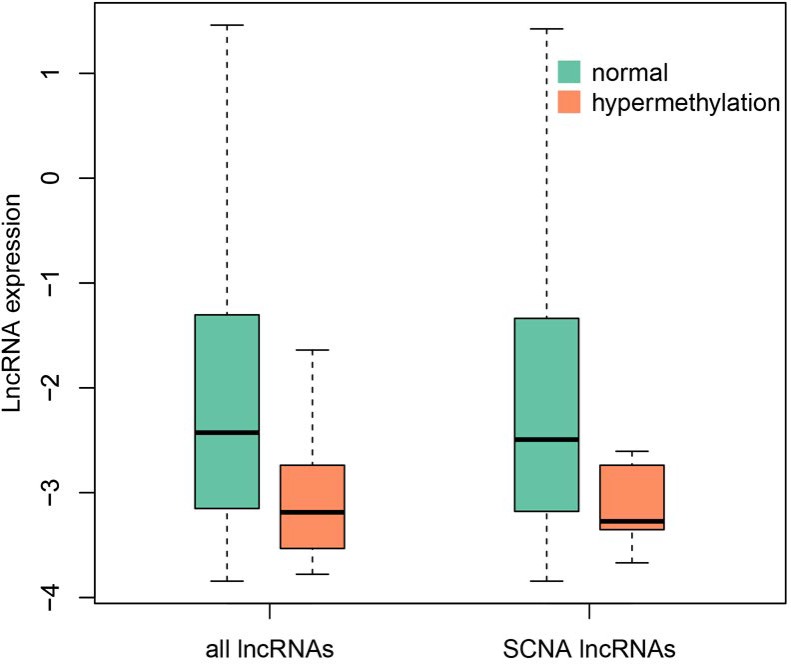
The expression comparison between hypermethylated lncRNAs and others The green represents the normal lncRNAs that expressed in LUAD, the orange represents the hypermethylated lncRNAs (Wilcoxon test, *p*-value = 0.0022 and 0.015, respectively).

In order to evaluate the impact of methylation on SCNA lncRNAs, we identified differentially methylated lncRNAs in promoter regions by *t*-test method [32] (Supplementary Data S3; 66 hypermethylation and 2 hypomethylation lncRNAs, respectively). 22 of 66 hypermethylation lncRNAs were expressed in LUAD. Furthermore, the expression of 22 lncRNAs was lower than that of the others in LUAD (Wilcoxon test, *p* = 0.0022). 17 hypermethylation lncRNAs occurred SCNA at the same time and had low expression (Wilcoxon test, *p* = 0.015, Figure 4). To explore the relationship between low expression and hypermethylation, we calculated PCC for each lncRNA. In total, 12 of them were negatively correlated (*p* < 0.05, Supplementary Data S4). These results implied that the expression of the 12 lncRNAs was affected by both of the copy number and methylation level in promoter regions.

### Cis-acting network

**Figure 5:**
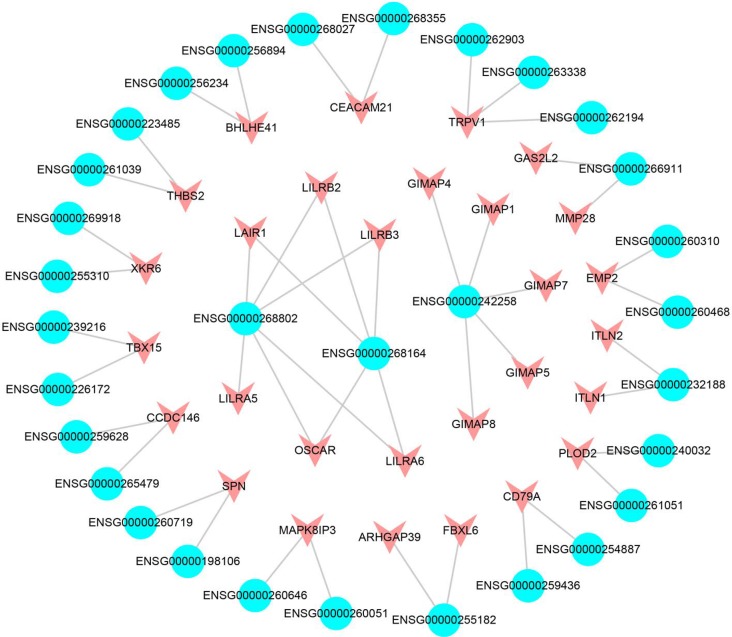
The cis-acting subnet of SCNA lncRNAs The blue represents the lncRNAs, the red represents the differentially expressed PCGs.

Regulatory lncRNAs can influence or interact with nearby or distant genes in both cis-acting regulation and trans-acting regulation [33, 34]. We first constructed a cis-acting network based on co-expression SCNA lncRNAs and differentially expressed PCGs pairs. In total, we obtained 146 pairs, including 130 SCNA lncRNAs and 128 differentially expressed PCGs (Supplementary Figure S2). The result exhibited that several lncRNAs significantly regulated multiple neighbor PCGs. One explanation for the phenomenon was that we selected differentially and significantly co-expressed PCGs. The ENSG00000268802 and ENSG00000268164 shared the same 5 PCGs (LAIR1, LILRB2, LILRB3, LILRB6 and OSCAR), and 4 of them belonged to leukocyte immunoglobulin-like receptor family (Figure 5). A study pointed out that LILRB2 was a receptor of natural killer (NK) cell [35] and was associated with lung cancer cell [36]. NK cells were the major subset of innate lymphoid cells and the first line of defense of infection endowed with complex regulatory roles [37]. In addition, NK cells were associated with the angiogenesis of LUAD [38]. OSCAR coded osteoclast-associated immunoglobulin-like receptors and related to immune mechanism [39]. Overall, these results revealed that lncRNA ENSG00000268802 and ENSG00000268164 may function by regulating the 5 target genes in cis-acting regulation. Furthermore, we found that the DSS of lncRNA ENSG00000268802 and ENSG00000268164 was low (0.03537 and −0.0188, respectively), which further confirmed our hypothesis that the low-DSS lncRNAs mainly took part in immune-related process.

The function of lncRNAs in network could be explored by further analysis of cis-acting regulation. For example, the lncRNA ENSG00000261685 (gene symbol: RP11-401P9.4) occurred copy number deletion and down-regulated in LUAD. The result demonstrated that the nearest co-expression PCG NKD1 occurred down-regulation at the same time [40]. NKD1 was an established transcriptional target of β-catenin complexes and a marker of aberrant Wnt/b-catenin signaling [41, 42]. A study revealed that the down-regulation of NKD1 increased the invasive potential of NSCLC and correlated with a poor prognosis [43]. Moreover, the down-regulated ENSG00000259974 regulated the target PCG FOXA2 that acted as a suppressor of lung cancer in cis-acting regulation [44]. FOXA2 was a key regulator for LUAD metastasis and down-regulated in LUAD [45]. The neighboring co-expression PCG MYEOV up-regulated by the overexpression of the amplification lncRNA ENSG00000260877. MYEOV functioned as an oncogene and overexpressed in many cancers including LUAD [46]. PCG CLDN4, the neighboring co-expression gene of ENSG00000225969 up-regulated in LUAD. A research indicated that the overexpressed CLDN4 was related with LUAD carcinogenesis [47]. These results showed that the SCNA lncRNAs played an important role in the occurrence, development and metastasis of lung cancer through cis-regulating neighboring target PCGs.

### The construction of lncRNA-TF-PCG triples

**Figure 6:**
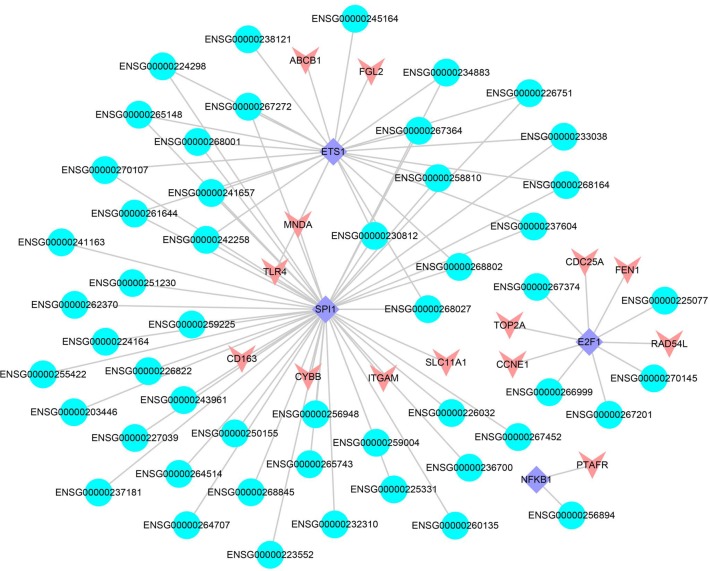
The lncRNA-TF-PCG network The blue represents the lncRNAs, the red represents differentially expressed PCGs and the purple represents the TFs.

The interactions between lncRNAs and TFs can improve the expression level of their target genes [34]. We obtained 245 LUAD-related lncRNA-TF-PCG triples, including 51 lncRNAs, 4 TFs and 14 differentially expressed PCGs, respectively (FDR < 0.05, Figure 6). 4 TFs (ETS1, SPI1, E2F1 and NFKB1) were the core of the trans-acting network and played important roles in cancer. For example, one of the TFs, ETS1, was related with the poor prognosis of LUAD [48]. Another TF, E2F1, promoted the apoptosis and acted as tumor suppressor [49]. Especially, the target PCGs of the 4 TFs were involved in some key cancer processes according to previous studies. Take CYBB, the target PCG of TF SPI1 as an example, it was found to be related with the prognosis of LUAD [50].

Furthermore, one of the major challenges of lung cancer immunotherapy was the tumor immunogenicity and immune escape. The PCG TLR4 that identified by us took part in trans-acting triples and provided clues for the inhibition of immune escape [51]. In the trans-acting network, 35 SCNA lncRNAs regulated TLR4 *via* TF SPI1 and ETS1 and formed a complicated subnet. There were 33 lncRNAs that the dosage sensitivity score of which was lower than 0.3, and 29 lncRNAs that the dosage sensitivity score of which was lower than 0.1 (Supplementary Figure S3). Combining the enrichment result that mentioned above, we could infer that the lncRNAs which participated in trans-acting regulation may affect cancer progresses *via* immune system. In a word, trans-acting network was helpful for providing key insights into tumor development and immune therapy.

### Functional prediction of lncRNAs based on lncRNA-function network

**Figure 7:**
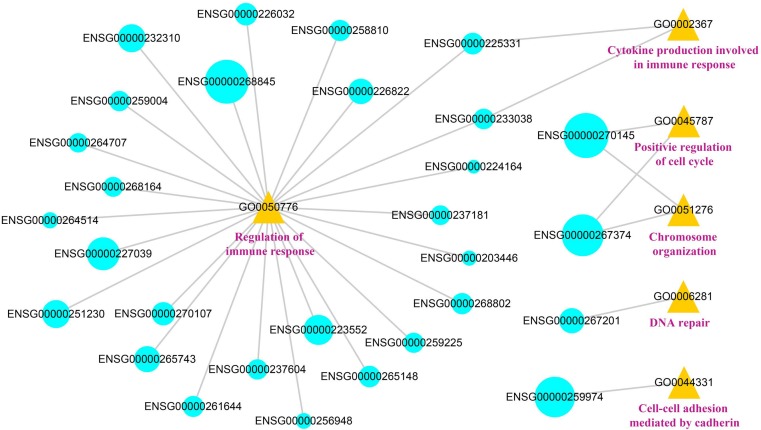
The visualization of the lncRNA-function network The blue represents the lncRNAs, the yellow represents the GO number and the pink represents the GO terms. The size of the blue circles represents the DSS of the lncRNAs.

Combining the results of cis-acting analysis and lncRNA-TF-PCG triples, we found that 10 lncRNAs involved in both cis-acting mechanism and trans-acting mechanism. It was suggested that the lncRNAs could function through different patterns. In order to provide deeply insights into the influences of SCNA lncRNAs in cancer, we obtained 171 lncRNAs from the cis-acting analysis and lncRNA-TF-PCG triples, and searched the GO terms through their target genes. We identified the lncRNAs that was significantly enriched in GO terms by hypergeometric test and constructed lncRNA-function network (FDR < 0.05). The network contained 29 lncRNAs and 6 GO terms (Figure 7). We found that the DSS of most of the lncRNAs that were related with immune regulation was lower than 0.5, while the score of lncRNAs that were related with cellular process was high. For example, the DSS of the ENSG00000270145 and ENSG00000267374 was 1.016 and 0.8857 respectively, and they involved in the process of cell cycle. The results further confirmed the functions of lncRNAs with different scores. The 29 lncRNAs that were related with immunoreaction in functional network may serve as candidate targets for LUAD immunotherapy.

### The identification of core regulation lncRNAs *via* ceRNA network

Recent studies verified that miRNAs was useful for lncRNA analysis [52–54]. For example, lncRNAs could act as miRNA sponges to regulate the expression of other transcripts [55]. In order to evaluate the ceRNA interactions that were mediated by lncRNAs, we constructed the lncRNA-miRNA-mRNA ceRNA network through a multi-step method (Supplementary Figure S4). First, we selected the PCGs that deregulated in LUAD and the lncRNAs that occurred SCNA. Second, the PCC value (*r* > 0.5, FDR < 0.05) was used as the strict threshold to identify significant lncRNA-PCG pairs. In addition, if both PCG and lncRNA in the same triple were co-expressed negatively with a certain common miRNA (*r* < 0, FDR < 0.05), this triple was remained for further selection. Finally, a hypergeometric test was performed to evaluate the significance of shared miRNAs for each possible pair (FDR < 0.05).

**Figure 8:**
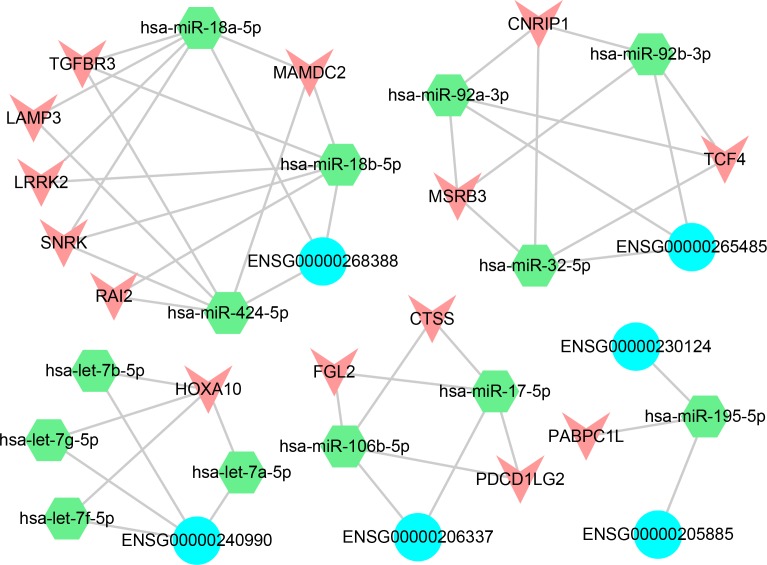
ceRNA network The blue represents the lncRNAs, the green represents the miRNAs and the red represents the differentially expressed PCGs.

All the functional pairs were integrated to form a miRNA-mediated lncRNA-associated ceRNA network, and we obtained 36 significant ceRNA pairs, including 6 lncRNAs, 13 miRNAs and 14 differentially expressed PCGs (Figure 8). The results showed that the lncRNAs competed with multiple PCGs to absorb miRNAs and formed a complex competition network. Take lncRNA ENSG00000268388 (FENDRR) as an example, it competed with 6 PCGs to absorb 3 miRNAs and formed a complex competition subnet. It was suggested that these lncRNAs may play key roles in the regulation of cancer processes. On the other hand, the PCGs that competed with lncRNAs in these subnet also had important functions. For example, PCG CTSS, FGL2 and PDCD1LG2 (PDL2) competed with ENSG00000206337 (HCP5) and formed a subnet. Previous study indicated that FGL2 may serve as a therapeutic target or a biomarker for tumor early detection [56]. Chen *et al.* found that PDL2 was an immune checkpoint gene [57]. In addition, a study pointed out that the lung cancer risk sites were significantly enriched in lncRNA HCP5 [58]. It was suggested that HCP5 may involve in the process of lung cancer by competing with PDL2 and FGL2.

### The prognosis ability of the ceRNA network

**Figure 9:**
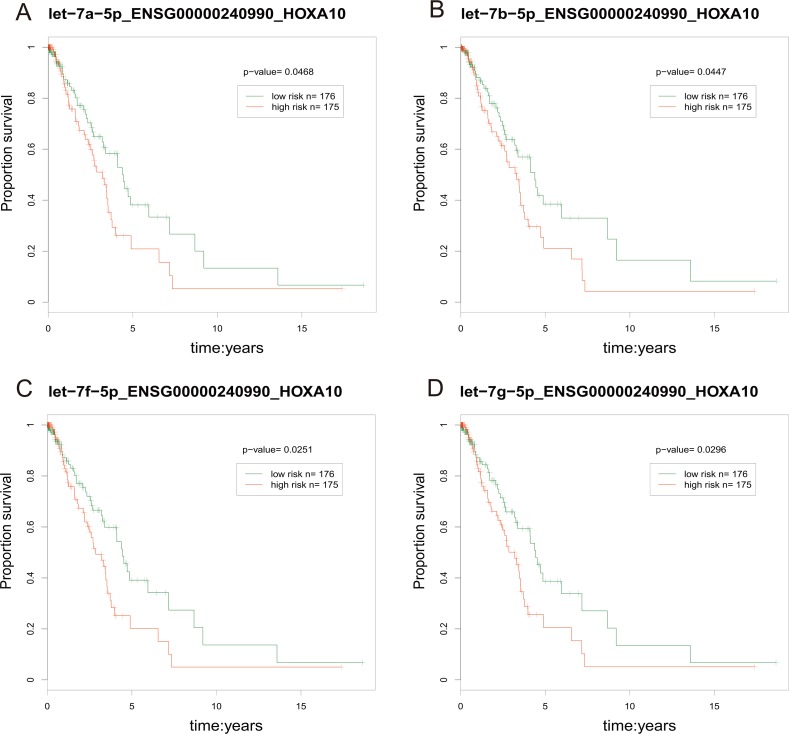
The prognosis-related ceRNA pairs The red line represents high-risk groups and the green line represents the low-risk groups. Samples with low risk were found to have longer survival.

We calculated the risk score [59] for each lncRNA-miRNA-PCG triple based on the risk score of single factor regression analysis of each node, and divided patient samples into high-risk and low-risk groups by median. The result showed that the ENSG00000240990-HOXA10 pair interacted with 4 miRNAs (hsa-let-7a/b/f/g-5p) and was significantly associated with prognosis (*p* < 0.05, Figure 9). The high-risk group had poor prognosis. Furthermore, the risk parameters of the 4 miRNAs were negative, while the risk parameters of the lncRNA ENSG00000240990 (HOXA11-AS) and PCG HOXA10 were positive. It was suggested that the patient survival can be affected by the ceRNA pairs. HOXA11-AS was a highly conserved lncRNA that located in the chromosome 7p15.2 [60]. Pervious study indicated that HOXA11-AS could serve as a biomarker for lung cancer metastasis and poor prognosis [61]. In our study, lncRNA HOXA11-AS and PCG HOXA10 were highly expressed (fold-change = 1.641 and 4.683, respectively). A study demonstrated that PCG HOXA10 overexpressed in lung cancer frequently [62] and was involved in the pathogenesis of lung cancer [63]. For lncRNA HOXA11-AS, it overexpressed in LUAD and was involved in the processes of NSCLC through regulating its target genes [64].

### Potential small molecule drugs for LUAD treatment

**Figure 10:**
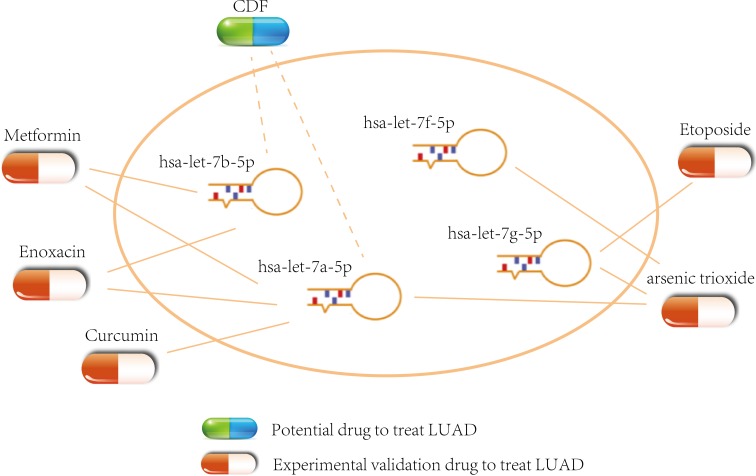
Potential small molecule drugs for LUAD treatment The capsules represent the experimental validation drugs and potential drug respectively.

In the ceRNA network, the perturbation of miRNA expression can influence the expression level of lncRNAs and PCGs [65, 66]. Therefore, we combined the information that was provided by SM2miR [67] with the module to infer potential small molecule drugs for LUAD treatment. In the ceRNA module, some potential drugs could up-regulate the hsa-let-7a/b/f/g-5p expression and further down-regulate the expression of related lncRNAs/PCGs and contribute to the treatment of LUAD (Figure 10). For example, curcumin could induce the apoptosis of cell NCI-H460 in NSCLC [68] and inhibit the transfer and invasion of lung cancer cell [69, 70]. In LUAD A549 cell, arsenic trioxide could induce the death of apoptotic cell [70]. Metformin could inhibit the growth of LUAD cell *via* apoptosis-inducing [71] and improve the survival of NSCLC patients in the stage IV [72]. In addition, we found other small molecule drugs that could regulate the 3 miRNAs such as CDF (analogues of curcumin). The effectiveness of these small molecule drugs could be validated by further experiment (Supplementary Data S5).

## DISCUSSION

According to the central dogma, the aberrance of copy number may affect cell functions. However, the SCNA could not directly function on cancer. It contributed to the pathway dysfunction by regulating gene expression [12]. Previous study demonstrated that SCNA was one of the most important mechanisms for lncRNA deregulation [13]. In order to discover the expression pattern of lncRNAs that affected by SCNA, we highlighted the differences of lncRNAs through matching SCNA profile and lncRNA profile, then selected dosage effect lncRNAs and analyzed the functions of lncRNAs with different DSS.

Our method that calculated DSS could well response to the variation tendency of the lncRNA expression and avoid noise. We set different threshold values to obtain DSS matrixes and found the similarity among these matrixes. The tendency consisted with our previous work [28]. It was suggested that fine-tuned parameters did not cause the remarkable influences. We selected different DSS thresholds for lncRNA classification and performed functional enrichment with DAVID. Notably, the lncRNAs with similar DSS involved in the same functions, while others played different roles in cancer biology. The downside of the analysis was that the lncRNAs with different dosage sensitivity may target the same PCGs. Therefore, we could not distinguish the function of lncRNA with DSS thresholds directly. Further functional validation of the lncRNAs with different DSS can be confirmed by experiment.

Accumulated evidence pointed out that the deregulation of lncRNAs was one of the driving factors for cancer [40, 73–75]. The two lncRNAs, ENSG00000259974 (LINC00261) and ENSG00000268388 that occurred copy number delection were down-regulated in our study. White *et al.* indicated that the two lncRNAs were associated with lung cancer [76]. In order to figure out the functions of SCNA lncRNAs in LUAD, we performed strict approach to analyze the lncRNAs based on lncRNAs-neighboring PCGs interactions and lncRNA-TF-PCG triples. Published medical literature showed that these lncRNAs were associated with the development and progression of lung cancer. For example, ENSG00000225969 could regulate the expression of CLDN4 by cis-regulation and lead to the carcinogenesis of LUAD. Further, we matched these lncRNAs to specific functions by cancer markers and screened out candidate targets that related with immunoreaction. These results may provide clues for LUAD immunotherapy.

We used strict approach to construct ceRNA network. The approach not only considered the lncRNAs/PCGs that acted as miRNA sponges, but also selected negative miRNA-target interactions. In addition, we provided the potential small molecule drugs that regulated the ceRNA network. Some small molecule drugs, such as Enoxacin and Etoposide, regulated the competitive relations of lncRNA-PCG pairs by influencing the expression of hsa-let-7a/b/f/g-5p, which provided novel clues for LUAD treatment.

Previous study solely focused on the analysis of single lncRNA and lacked systematic research. In order to analyze the function of lncRNAs in 3 regulatory network, we constructed lncRNA-function network and mapped each lncRNA to matched GO terms. This results increased the understanding of lncRNAs and provided reference for functional research. LncRNAs could serve as cancer biomarker because of their tissue specificity [77]. Combining the expression of functional lncRNAs with the clinic information of LUAD patients, we identified prognosis-related ceRNA pairs. In conclusion, these results will help understand the development and progression of SCNA lncRNAs in cancer and provide deeply insights into the cancer biological mechanism.

Similar to other computational approaches, our study also had some limitations. First, most of the lncRNAs that mapped to the copy number delection regions did not have expression value or have low expression in LUAD, leading to the missing of SCNA lncRNAs. Second, we used cancer marker-related GO terms to construct lncRNA-function network, while these GO terms only represented a small fraction. Last, for survival analysis, because of lacking other clinic data that matched with ours, it was hard to further validate prognosis-related lncRNA. These deficiencies would be improved by the complement of data in future.

## MATERIALS AND METHODS

### Data and pre-processing

The RNA-seqV2 data, SCNA profile and clinic information of LUAD were downloaded from The Cancer Genome Atlas (TCGA). The RSEM values of PCGs were normalized by upper quartile. We obtained an expression matrix that contained 576 patient samples (59 normal samples and 517 tumor samples) and 20,513 PCGs by combining the RNA-seqV2 data. PCGs with missing values in > 30% of the samples were removed. In total, we obtained 15,149 PCGs. Moreover, we added 2 to the expression value of each PCG and performed log2-transformed. The lncRNA profile of LUAD was obtained from TANRIC database. The lncRNAs that either 50% quantile = 0 or 90% quantile≤0.1 were removed. We added 0.05 to the expression value of each lncRNA and performed log2-transformed. Finally, we obtained 5,109 lncRNAs and 546 patient samples (58 normal samples). The hg19 (GRCh37) comprehensive gene annotation and lncRNA annotation were downloaded from GENCODE database.

### Combining linear and nonlinear regression to calculate dosage sensitive score

First, we considered the samples with SCNA values in [−0.3, 0.3] as normal ones, which meant that the samples had no SCNA. Second, in order to summary the tendency between lncRNA expression and SCNAs, the lncRNAs that had SCNA in more than 20% samples were selected for further analysis. Considering SCNA value and expression value as variable, we evaluated the effect of SCNA on lncRNAs. The method of calculating DSS [28] that we selected took more information into consideration and was more accurate than the method that based on samples. The higher score of the DSS of PCGs or lncRNAs was, the stronger their dosage sensitivity was.

### DNA methylation

DNA methylation data of LUAD from Illumina Infinium Human Methylation 450 Beadchip (Infinium 450k) arrays was obtained from TCGA, including 485 577 probes, 475 tumor samples and 32 normal samples. We then mapped cg probes to promoter regions. The promoter regions were defined as the regions 2kb upstream from the TSSs [78]. The probes that mapped to more than one promoter regions were removed. The missing values were replaced by average values. If multiple probes were mapped to the same lncRNA, the average value was considered as the methylation beta value of the lncRNA. The *t*-test was performed to identify the differential methylation genes (fold change > 2, FDR < 0.01). The fold change value of each gene was calculated by the ratio of the average methylation beta value of tumor samples and normal samples.

### Differential expression analysis

We performed SAM algorithm to identify differentially expressed genes based on PCG profile and lncRNA profile. For each PCG or lncRNA, the R samr package [79] was used for selecting differentially expressed genes (fold change > 2). The Benjamini-Hochberg (BH) method was used to calculate FDR value (FDR < 0.05). In total, we obtained 3,654 differentially expressed PCGs (2,185 down-regulated PCGs and 1,469 up-regulated PCGs) and 608 differentially expressed lncRNAs (382 down-regulated lncRNAs and 426 up-regulated lncRNAs).

### LncRNA-PCG co-expression

The Pearson Correlation Coefficient (PCC) was used to calculate the co-expressed coefficient between SCNA lncRNAs and differentially expressed PCGs (BH method, FDR < 0.05, *r* > 0.5).

### LncRNA-miRNA interaction pairs

The lncRNA-miRNA and mRNA-miRNA interaction pairs were obtained from starBase v2.0. After removing redundancy, we obtained 10,112 lncRNA-miRNA interaction pairs, including 132 miRNAs and 1,114 lncRNAs, and 323,648 mRNA-miRNA interaction pairs, including 366 miRNAs and 12,386 mRNAs.

### Cis-acting and trans-acting regulation prediction

The expression profiles of lncRNAs and PCGs were used to predict the cis-acting regulation and trans-acting regulation in our study [33, 34]. For cis-acting regulation, we recognized coupled genomic locations of lncRNAs and PCGs, the neighboring genes within 300kb of the lncRNAs were considered as potential cis-acting targets of lncRNAs.

LncRNAs may function *via* TFs in trans-acting regulation. First, we obtained experimentally verified TF-gene interaction pairs from TRED. Second, PCC value was used to select the TFs that co-expressed with SCNA lncRNA (FDR < 0.05, *r* > 0.6). Finally, for each given lncRNA, we calculated the overlaps between co-expressed PCGs and the target genes of a given TF. The hypergeometric distribution method was performed to test the significance of the overlaps. If the co-expressed PCGs of a given lncRNA were significantly overlapped with the target genes of a given TF, the TF may interact with the lncRNA, and these PCGs may be regarded as the trans-acting target of the lncRNA.

### The construction of ceRNA-ceRNA network

The intersection of co-expression lncRNA-PCG pairs and lncRNA-miRNA interaction pairs was used to identify ceRNA pairs. A hypergeometric test was performed to evaluate the significance of shared miRNAs for each possible gene pair:

$$P=1-\sum_{i=0}^{x-1}\frac{\left(\begin{array}{c} L\\ i \end{array}\right)\left(\begin{array}{c} N-L\\ M-i \end{array}\right)}{\left(\begin{array}{c} N\\ M \end{array}\right)}$$

Where N is the total number of miRNAs which were interacted with lncRNAs or PCGs, M is the number of miRNAs interacting with this given lncRNA, L is the number of miRNAs interacting with this given PCGs, and x is the number of miRNAs that interact with both of them, respectively. The P-value and FDR correction less than 0.05 were used as the threshold. Finally, the significant ceRNA pairs were used to construct ceRNA-ceRNA network. We used Cytoscape v3.1 to visualize the network and analyze the topological property of network.

## SUPPLEMENTARY MATERIALS FIGURES AND TABLES













## Abbreviations

SCNA: somatic copy number alternation
DSS: dosage sensitivity score
LUAD: lung adenocarcinoma
NSCLC: non-small cell lung cancer
PCGs: protein-coding genes
