# Supplementary material for: Integrated analysis of dosage effect lncRNAs in lung adenocarcinoma based on comprehensive network
Source: Oncotarget. 2017 Aug 3;8(42):71430–46. doi: 10.18632/oncotarget.19864 (PMC5641060; doi:10.18632/oncotarget.19864)
Supplement: Supplementary file 1 [file oncotarget-08-71430-s001.pdf]

Integrated analysis of dosage effect lncRNAs in lung adenocarcinoma based on comprehensive network

SUPPLEMENTARY MATERIALS

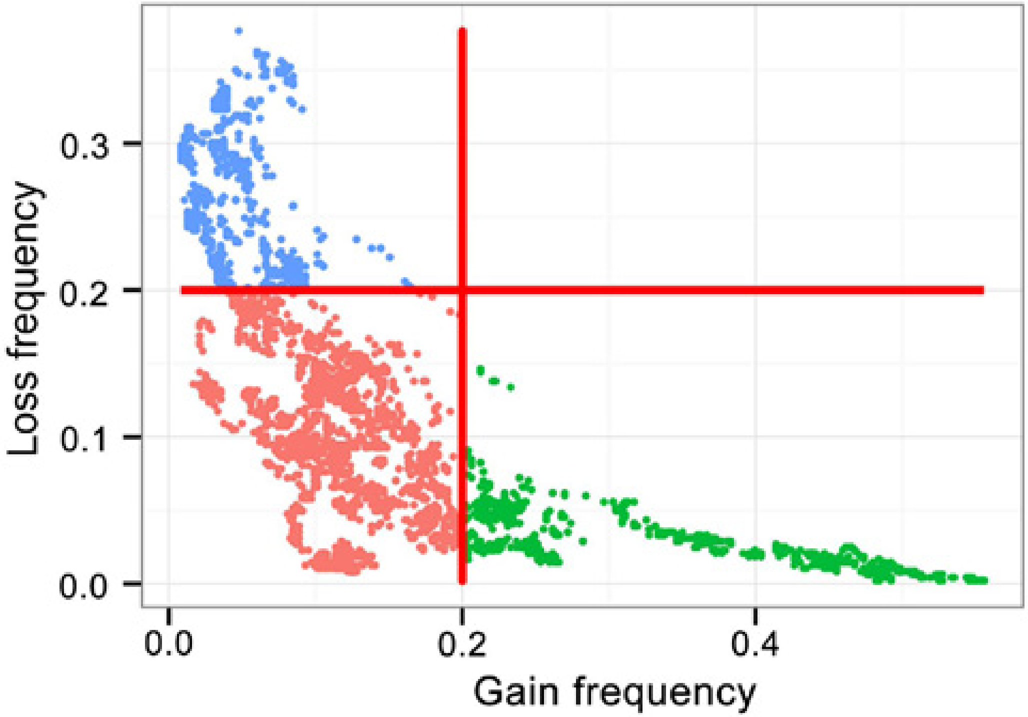

Supplementary Figure S1: The gain/loss frequency of lncRNAs across tumor samples.

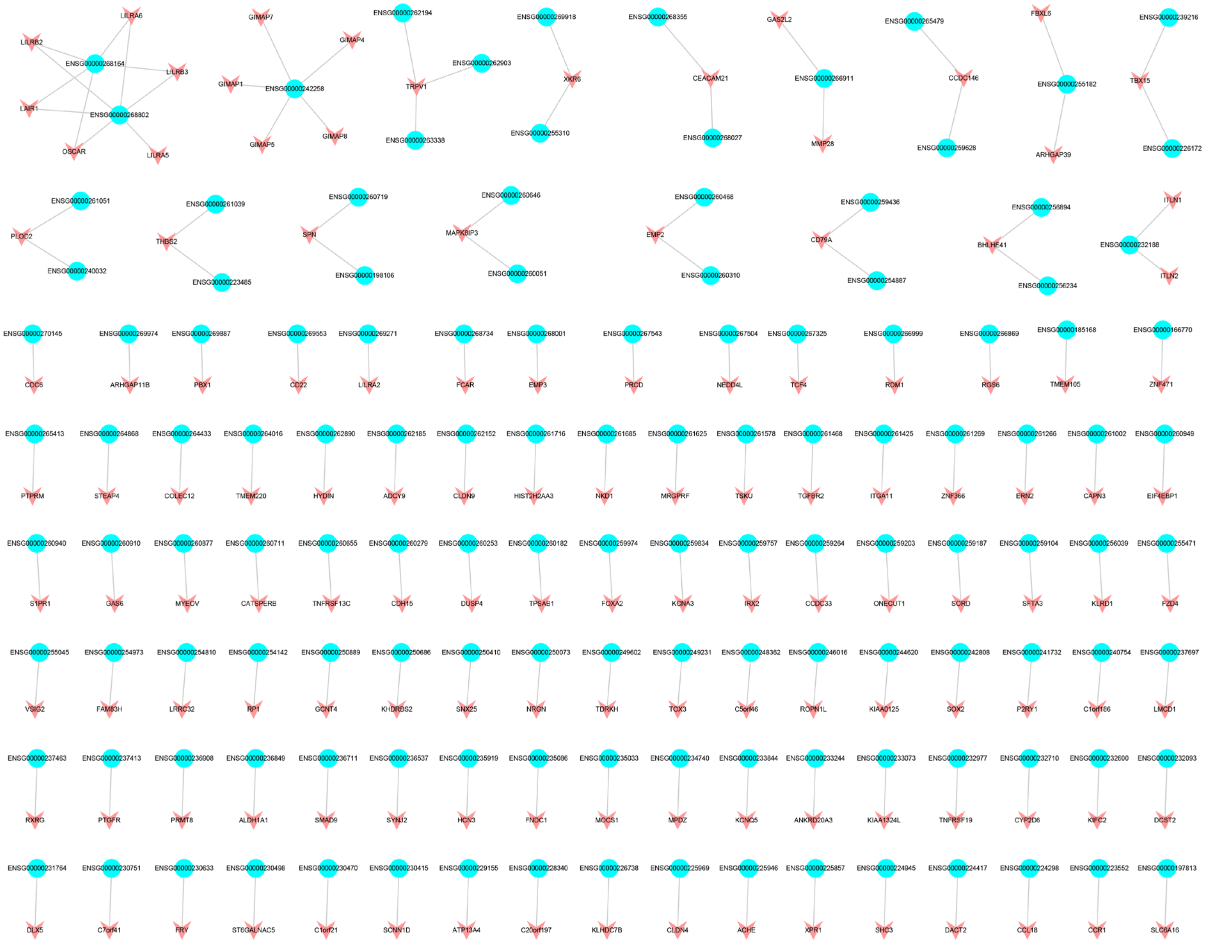

Supplementary Figure S2: The cis-acting network of SCNA lncRNAs.

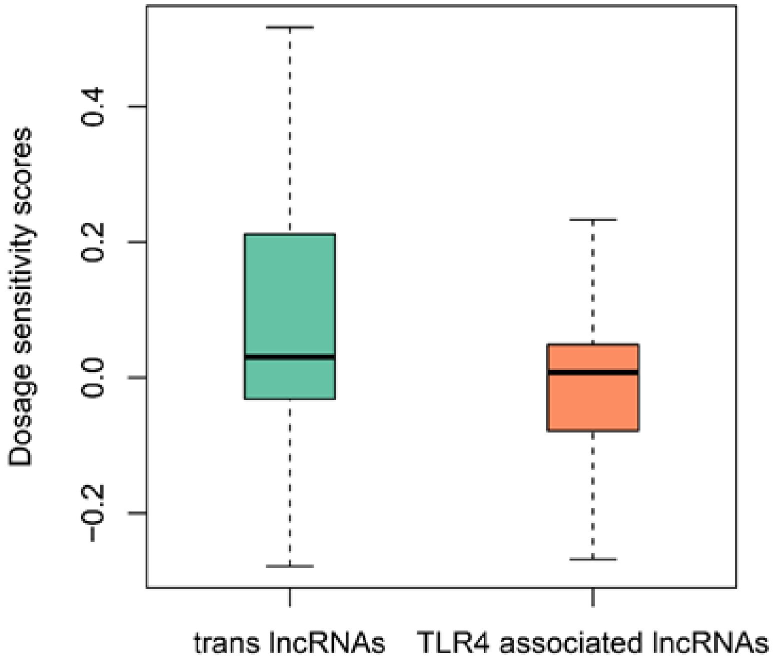

Supplementary Figure S3: The DSSs of SCNA lncRNAs involved in trans-acting network.

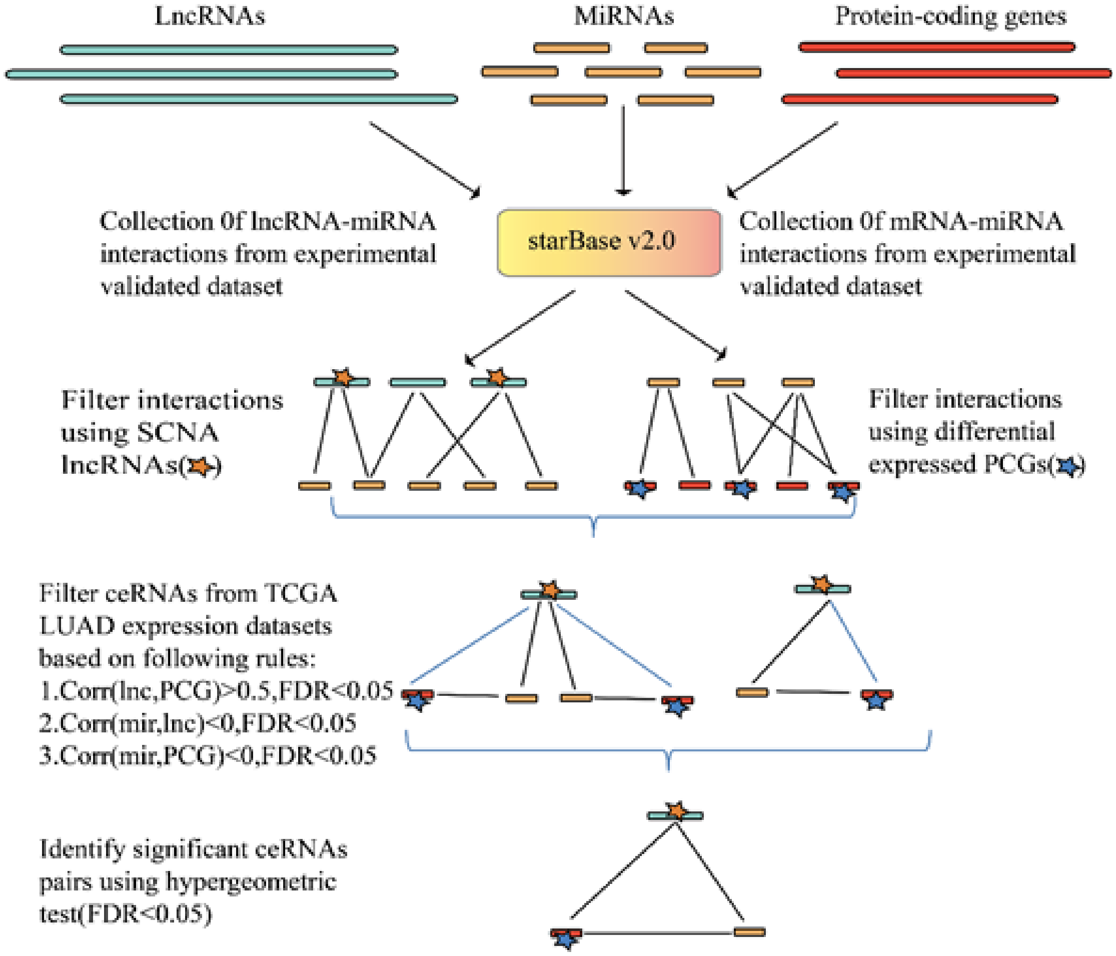

Supplementary Figure S4: Pipeline for transcriptome-wide identification of significant LUAD-related ceRNA pairs.

For Supplementary Tables see in Supplementary Files

Supplementary Data S1: 179 amplified and 881 deleted lncRNAs.
Supplementary Data S2: 3,499 SCNA lncRNAs.
Supplementary Data S3: 66 hypermethylation and 2 hypomethylation lncRNAs.
Supplementary Data S4: The PCC between methylated value and expressed value of 17 SCNA lncRNAs.
Supplementary Data S5: List of the potential small molecule drugs.
